# ^1^H NMR Profiling of Honey Bee Brains across Varying Ages and Seasons

**DOI:** 10.3390/insects15080578

**Published:** 2024-07-30

**Authors:** Nuria Morfin, Paul H. Goodwin, Ernesto Guzman-Novoa, Nicole Legge, James Longstaffe

**Affiliations:** 1 School of Environmental Sciences, University of Guelph, Guelph, ON N1G 2W1, Canada; pgoodwin@uoguelph.ca (P.H.G.); eguzman@uoguelph.ca (E.G.-N.); jlongsta@uoguelph.ca (J.L.); 2 The British Columbia Technology Transfer Program, British Columbia Honey Producers’ Association, P.O. Box 5594, Station B, Victoria, BC V8R 6S4, Canada

**Keywords:** *Apis mellifera*, honey bee, metabolome, aging, nuclear magnetic resonance, NMR

## Abstract

**Simple Summary:**

Metabolic changes in adult honey bee brains associated with the seasons and aging in honey bees (*Apis mellifera*) were assessed using nuclear magnetic resonance (NMR) spectroscopy. Brains were dissected from newly emerged, 14-day– old, and 28-day– old bees that were collected over the summer, as well as brood nest bees collected in the fall, winter, and spring. Changes in the brain metabolome indicated that worker bees undergo adaptations throughout their lives, with notable shifts occurring between newly emerged and older individuals. Furthermore, the seasons affected the honey bee brain metabolome, highlighting differences in metabolic responses to changes in the environment and resource availability. While previous studies have reported differences in the metabolome of whole bodies between summer and winter bees, or in the brains of forager bees infected with a virus, the present study has revealed the metabolome of the honey bee brain as it ages. Several potential markers were identified for assessing aging of the bee brain, such as the potential neurotransmitter β-alanine and neurotransmitter-precursors/intermediates, phenylalanine, tyrosine, tryptophan, and O-phosphocholine.

**Abstract:**

Honey bees (*Apis mellifera*) provide a useful model for studying aging because of the differences in longevity between the relatively short-lived summer and long-lived winter bees, as well as bees lacking signs of cognitive senescence as they age. Bee brains were dissected from newly emerged, 14-day–, and 28-day– old bees in mid- and late summer, as well as brood nest bees in fall, winter, and spring, before, during, and after overwintering, respectively. Brains were examined with nuclear magnetic resonance (NMR) spectroscopy to analyze their metabolome. Nine variable importance in projection (VIP) variables were identified, primarily amino acids and choline derivatives. Differences in metabolite concentrations were found with different ages of summer bees, mostly between newly emerged and 14-day– old bees, such as a decrease in phenylalanine and an increase in β-alanine, but there were also changes in older adults, such as o-phosphocholine that declined in 28-day– old bees. Differences in brood nest bees were observed, including a decline in tryptophan and an increase in β-alanine. These may provide distinct metabolomic signatures with age and season. Such research holds promise for a better understanding of the complex interplays between bee physiology, development, and aging, which has implications for improving bee health and management.

## 1. Introduction

Honey bees (*Apis mellifera* L.) play an important role in ensuring global food security as well as in sustaining agroecosystems [1]. However, honey bees have suffered high overwinter colony losses in recent years worldwide, mostly in the Northern Hemisphere where colonies can be stored in winter facilities for several months [2,3]. High overwinter colony losses could be related to the effects of multiple stressors, including pathogens, poor nutrition, and extreme climatic conditions [4,5,6]. Biomarkers that could help predict overwinter colony losses are needed and some have been proposed, including the expression levels of immune-related genes and the abundance of different viruses [7,8]. However, the use of metabolites as biomarkers may provide an alternative that could be more practical for screening large numbers of colonies.

Metabolomics, the study of metabolites in a tissue or organism [9], has been successfully utilized to identify biomarkers as diagnostic tools for several human diseases using biofluids, tissue extracts, and tissue biopsies [10]. Nuclear Magnetic Resonance (NMR) spectroscopy is a metabolomic technique that allows for the non-specific identification of metabolites [11]. This technique has been used for the identification of health biomarkers in animals such as dogs and cows [12] and has the potential to be used with another economically important animal, the honey bee, such as detecting proline as a marker in bee brains for the deformed wing virus infection [13]. Worker honey bees undergo physiological changes as they age and many of these changes are related to age polyethism [14]. In addition to this, the life span of honey bees is highly variable. In the spring and summer, worker honey bees live approximately five to eight weeks [15,16]. Initially, they spend two to three weeks as nest bees, remaining inside the hive and caring for the larvae and the queen. This is followed by approximately three to four weeks spent as foragers, collecting pollen and nectar from flowers, or acting as guard bees, among other tasks [17]. During the fall, winter bees are produced, and these bees can live up to six months throughout the winter [15,16]. After the winter, in spring and summer, bees live shorter lives again [15,16]. These differences in length of life in different seasons have been associated with lower levels of juvenile hormones and higher levels of vitellogenin in winter bees compared to summer bees [18,19], whereas summer bees have higher levels of juvenile hormones but lower levels of vitellogenin [14,18]. A recent study on the metabolic profile of whole bodies of summer and winter worker bees showed multiple differences between them, including higher levels of trehalose in winter bees [20], indicating that some metabolites are markedly changed between seasons, possibly due to changes in metabolic demands. However, that study was limited to a single time point for each season and the use of whole bodies rather than specific tissues, which could present a challenge due to the physiological changes that different organs undergo as they age in addition to the consequence of seasonal changes. Nonetheless, changes in metabolite concentration in healthy bees could be used to later evaluate the effect of stressors on honey bee health.

Honey bee brains undergo changes related to the tasks the insects perform as they age. For example, brains of older worker bees showed higher gene expression related to signal transduction and neurotransmitter transporters compared to younger worker bees, indicating greater neuronal activity in adult worker bees [21]. Older foraging bees also had lower levels of kinases, as well as synaptic and neuronal growth-related proteins in the central brain, which could be related to learning dysfunction [22]. Rapid aging in worker bees associated with foraging correlated with declines in glutamine synthetase and glycogen phosphorylase in the brain. These enzymes are involved in nutritional homeostasis and aging [23]. Additionally, winter and summer bees are differentially affected by aging, with winter bees not displaying age-related declines in cognitive processes, such as learning and memory, that are observed in summer bees [24]. Furthermore, oxidative stress, indicated by oxidative nitration and carbonylation damage in the brain of bees, was lower in winter bees compared to summer forager bees [25]. Thus, a number of aspects of the bee brain change with aging, and at least some of those aspects differ between the relatively long-lived winter and short-lived summer bees. In addition to providing biomarkers, an analysis of the metabolite composition of honey bee brain tissue will provide a baseline for comparison in future studies of factors that may impact cognition and the ability of bees to perform essential tasks, such as foraging.

The honey bee, as a eusocial organism, has been proposed as a good model for studying animal aging and cognition [26,27,28]. Given that the honey bee brain is altered while aging and that the effects of aging appear to differ between summer and winter bees, the aim of this study is to determine if differences occur in the metabolomic profile of the brains from healthy honey bees collected at different ages and during different seasons of the year, including summer foragers and bees that emerge just prior to overwintering. Differences in the metabolomic profiles of long- and short-lived bees may be useful for identifying markers related to longevity as an assessment of stressors on honey bee health.

## 2. Materials and Methods

### 2.1. Source of Worker Bees

Honey bees were obtained from colonies of the Buckfast strain at the Honey Bee Research Centre, University of Guelph, ON, Canada (N 43°3210:586″, W 80°12′49.736″) in 2019 and 2020. The queens of the colonies used for the study were mated under controlled conditions, in isolation at Thorah Island (Simcoe, ON), to guarantee the purity of the genotype. Additionally, the colonies used for the study were not previously exposed to pesticides and showed no visible signs of disease. To obtain bees from different ages (newly emerged bees, brood nest bees, and 14- and 28-day– old bees) and at different timepoints throughout the season (summer, fall, winter, and spring), the following schedule was used (Table 1).

Newly emerged bees (<24 h) from each colony were obtained from frames placed in incubation in screened emerging cages (50.3 × 7.3 × 25.2 cm) at 35 °C and 60% RH overnight. A total of 1500 newly emerged bees from each colony were collected and color marked on the thorax using a marker (POSCA, Uni Mitsubishi Pencil, Shinagawa, Japan). Each cohort of bees was marked with a different color to ensure the collection of bees at the appropriate age. After marking, the bees were returned to their respective hives. Worker bees were sampled at 1, 14, and 28 days after emergence. Additionally, brood nest bees were collected in September of 2019, February of 2020, and lastly May of 2020; a total of 50 worker bees from each colony and at each timepoint were collected and kept in dry ice to be transported to the laboratory where they were stored at −70 °C until analysis.

### 2.2. Brain Dissections

The brains of worker bees were dissected as per Morfin et al. [29]. Briefly, a No. 21 stainless steel surgical blade (Integra, Fisher Scientific, Mississauga, ON, Canada) was used to make a longitudinal incision through the exoskeleton of the epicranium to expose the brain. The brain was removed using forceps (120 × 8 × 17 mm; FisherBrand, Mississauga, ON, Canada) and stored at −70 °C until metabolite extraction. The brains of 15 worker bees from each colony were dissected and used for metabolite extraction.

### 2.3. Metabolite Extraction

Total metabolites from brain tissue were extracted as per Viant [30]. Briefly, each sample of approximately 100 mg, obtained from 15 worker bee brains, was homogenized and transferred into 15 mL conical centrifuge tubes and kept on ice during the extraction process. A total of 200 µL of molecular grade H_2_O, MeOH, and CHCl_3_ were poured into each of the 15 mL tubes (Fisher Scientific, Mississauga, ON), which were then vortexed for 20 s at 10,000 rpm. After that, the samples were placed in a water bath–sonicator for 10 min (Fisher Scientific, Mississauga, ON, Canada). Then, 800 µL of CHCl_3_ and 200 µL of molecular grade H_2_O were added and the samples were vortexed a second time for 20 s at 10,000 rpm, followed by a centrifugation at 14,000 rpm for 20 min at 0 °C (Thermo Scientific, Sorvall Legend Micro 21R, Mississauga, ON, Canada). Finally, 800 µL of supernatant (polar phase) was transferred to a new 1.5 mL microcentrifuge tube for each sample. The tubes were placed in a centrifugal evaporator at 15,000 rpm at 35 °C for 2.5 to 4 h (Savant SpeedVac, Fisher Scientific, Mississauga, ON, Canada). The samples were then kept at −70 °C until NMR analysis.

### 2.4. NMR Analysis

Samples were prepared for NMR analysis by reconstituting the dried metabolite extracted above in a 0.2 M D2O phosphate buffer prepared using sodium phosphate monobasic and sodium phosphate dibasic including 0.1% *w*/*v* sodium azide as a preservative [31]. The pH was corrected to 7.0 using minimal quantities of sodium deuteroxide. In addition, 3-(trimethylsilyl)-1-propanesulfonic acid sodium salt (DSS) was included as an internal chemical shift and concentration standard. All reagents were acquired from Sigma Aldrich. The 1D ^1^H nuclear magnetic resonance (NMR) spectra were obtained on a Bruker Avance III 600 MHz spectrometer equipped with a Bruker TCI cryoprobe (Bruker Biospin, Billerica, MA, USA). Spectra were acquired using NOESY-presaturation with 32 scans and 4 dummy scans with an optimized 90° pulse, a time domain of 96 k data points, and a sweep width of 17 ppm. A recycle delay of 4 s was employed. The spectra were processed using 262 K points and apodized using an exponential function equivalent to 0.5 Hz line broadening. The spectra were also processed using Chenomx (Edmonton, AB, Canada) to identify and quantify key metabolites.

### 2.5. Data Processing and Statistical Analysis

The data were subjected to a Shapiro–Wilk test to assess for normality and Levene’s test to assess homogeneity of variances. Metabolite concentrations (mM) were subjected to a principal component analysis (PCA) as a reduction technique. The data were processed using probabilistic quotient normalization, square root transformation, and Pareto scaling using MetaboAnalyst 6.0 [32]. A Partial Least Squares–Discriminant Analysis (PLS-DA) was used to identify variable importance in the projection scores (VIP). Metabolites with VIP value >1.0 were considered significant. Additionally, a Kruskal–Wallis test was conducted with ranked transformed data to compare metabolite concentrations when significance was detected, and multiple comparisons were performed using Conover–Iman procedures and Bonferroni corrections to confirm if age and season affected the concentration of metabolites in honey bee brains. Statistical analyses were conducted using XLSTAT 2023.2.1414 [33] with an α of 0.05.

## 3. Results

### 3.1. NMR Spectrum of Honey Bee Brains

**Figure 1:**
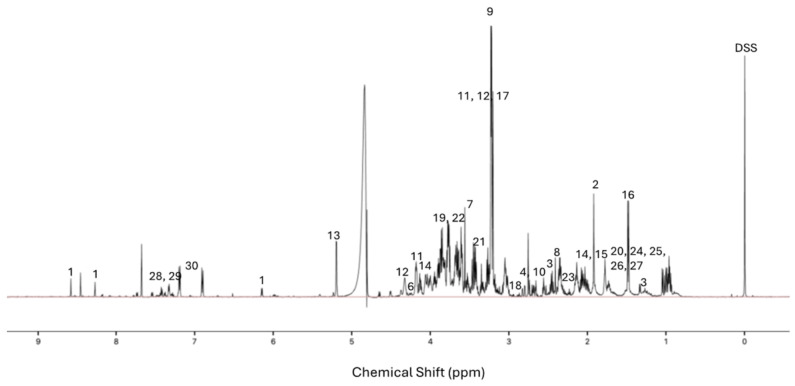
^1^H NMR spectrum of extracted bee brain metabolites. 1 AMP, 2 acetate, 3 suberate, 4 aspartate, 5 B-alanine, 6 threonine, 7 glycine, 8 succinate, 9 trimethyl-N-oxide, 10 sarcosine, 11 O-phosphocholine, 12 sn-glycero-3-phosphocholine, 13 trehalose, 14 proline, 15 glutamate, 16 alanine, 17 choline, 18 tyrosine, 19 glucose, 20 putrescine, 21 taurine, 22 fructose, 23 glutamine, 24 isoleucine, 25 leucine, 26 valine, 27 lysine, 28 phenylalanine, 29 tryptophan, 30 tyrosine, and DSS (3-(trimethylsilyl)-1-propanesulfonic acid sodium salt).

A total of 30 metabolites were identified in the NMR spectra, including 19 amino acids/amino acid intermediates or amino acid derivatives (alanine, aspartate, creatine, glutamate, glutamine, glycine, isoleucine, leucine, lysine, phenylalanine, proline, putrescine, sarcosine, taurine, threonine, tryptophan, tyrosine, valine and β-alanine), three choline derivatives (choline, O-phosphocholine and glycero-3-phosphocholine), three sugars (fructose, glucose and trehalose), two dicarboxylic acids (malonate and succinate), one carboxylic acid (acetate), one nucleotide (AMP), and one amine oxide (trimethylamine N-oxide) (Supplementary Table S1). A typical ^1^H NMR spectrum of the metabolites extracted from honey bee brains is shown in Figure 1.

### 3.2. Principal Component Analysis for Summer, Fall, and Winter Bees

**Figure 2:**
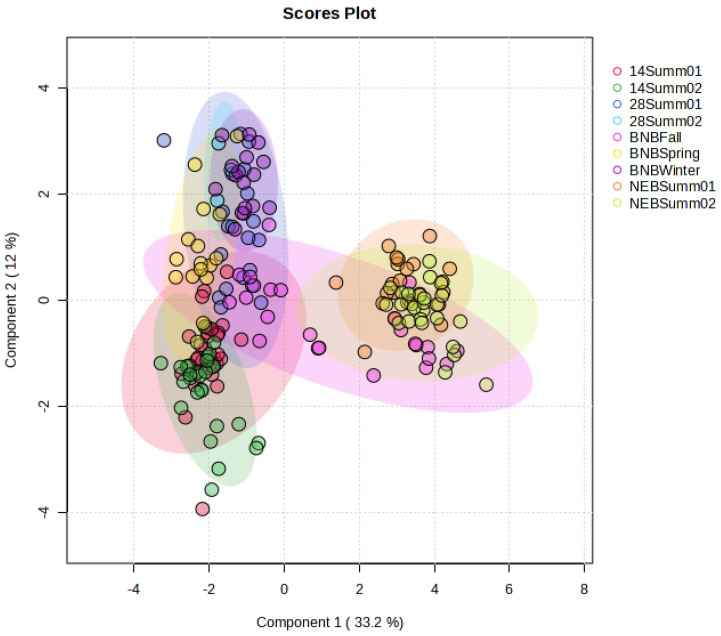
Principal component analysis of newly emerged bees collected in the summer (NEBSumm01 and NEBSummer02), 14– day-old worker bees collected in the summer (14Summer01 and 14Summer02), 28-day– old worker bees collected in the Summer (28Summer01 and 28Summer02), brood nest bees collected in the fall (BNBFall), winter (BCBWinter), and spring (BNBSpring). A score plot (a) PC1 on the *x*-axis explains 33.2% and PC2 explains 12% of the variability.

The principal component analysis (PCA) showed no separation between the newly emerged bees collected in the summer (NEBSumm01 and NEBSum02; Figure 2). Similarly, 14-day– old summer bees collected at the two timepoints were clustered together (14Summ01 and 14Summ02), with the same observation for the 28-day– old bees collected in July and August (28Summ01 and 28Summ02). A clear separation between newly emerged bees and adult bees (14- and 28-day– old bees) was observed; however, this separation was not observed between 14- and 28-day– old bees and brood nest bees collected in the fall, winter, or spring (BNBFall, BNBWinter, and BNBSpring). In fact, BNBWinter and BNBSpring overlapped with the 28-day– old bees, BNBSpring overlapped with the 14-day– old bees. The first two principal components explained 45.2% of the total variability in the data (PC1 = 33.2%, PC2 = 12%). These results emphasize the distinction in the brain metabolites between the newly emerged bees and adult bees.

### 3.3. Statistically Significant Metabolites

**Figure 3:**
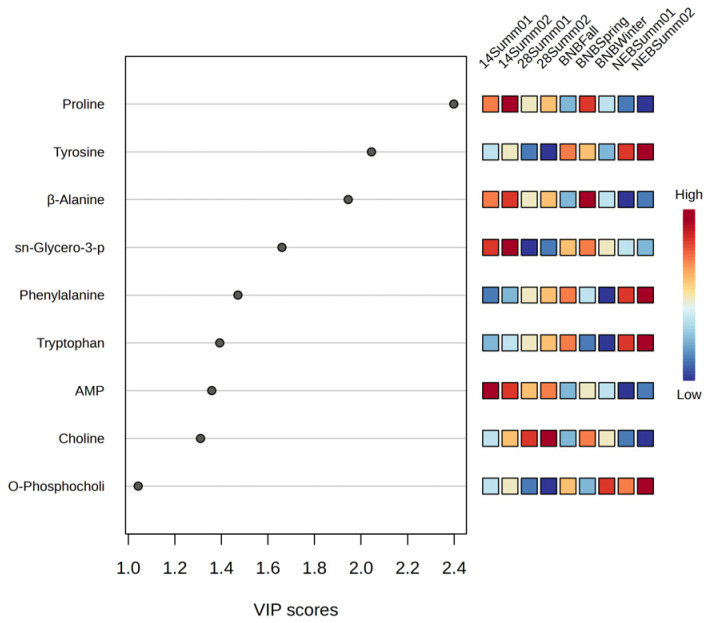
Nine identified variable importance in projection scores (VIP) > 1 (from highest to lowest). In the VIP plot the colored boxes indicate the relative intensities of the corresponding metabolites in 14-day– old bees and 28-day– old worker bees collected in July and August (14Summer01, 14Summer02, 28Summer01, and 28Summer02, respectively), brood nest bees collected in the fall, spring, and winter (BNBFall, BNBSpring, BNBWinter, respectively), and newly emerged bees collected in June and July (NEBSumm01 and NEBSumm02, respectively). Red represents higher relative abundance and blue represents lower relative abundance.

**Figure 4:**
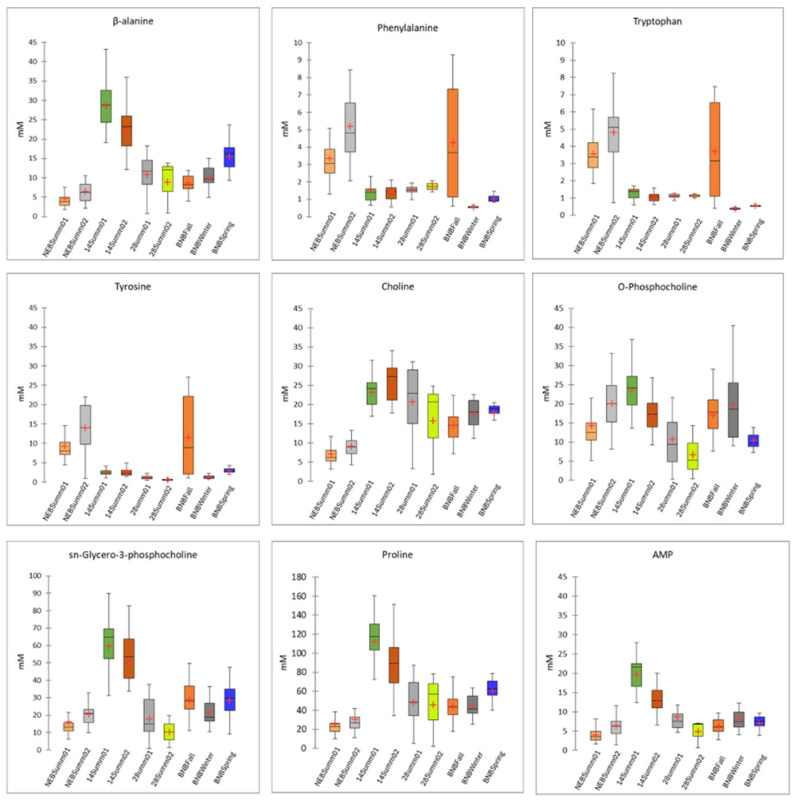
Box plots of metabolite concentrations (mM) of the nine VIP (>1): β-alanine, phenylalanine, tryptophan, tyrosine, choline, phosphatidylcholine, O-phosphocholine, sn-glycerol-3-phosphocholine, proline and AMP. Significant differences between newly emerged bees collected in June and July (NEBSumm01 and NEBSumm02, respectively), 14-day– old summer bees collected in July and August (14Summ01 and 14 Summ02, respectively), 28-day– old bees collected in July and August (28Summ01 and 28Summ02, respectively), and brood nest bees collected in the fall, winter or spring (BNBFall, BNBWinter, and BNBSpring, respectively) were calculated using Kruskal–Wallis tests and Conover–Iman procedures and Bonferroni correction. Pairwise comparisons using Conover–Iman procedures (adjusted *p* value of 0.0014) are shown in Supplementary Table S4. Non-transformed data are presented. In the box plots, the medians are shown by the horizontal lines inside the boxes, the 25th and 75th percentiles are shown as the bottoms and tops of the boxes, the minimum and maximum values are represented as the small horizontal lines below and above the boxes (outliers are not shown), the mean is represented by red cross (+).

Nine VIP > 1 scores were identified, which were for proline, tyrosine, β-alanine, sn-glycero-3-phosphocholine, phenylalanine, tryptophan, adenosine monophosphate (AMP), choline, and O-phosphocholine (Figure 3 and Supplementary Tables S2 and S3). Higher levels of phenylalanine were detected in the newly emerged bees collected in the summer (NEB Summ01 and NEBSumm02) and in the brood nest bees collected in the fall (BNBFall) (Figure 4 and Table S4). Significant differences in phenylalanine concentrations were observed among the newly emerged bees (NEB01 and NEB02), the 14-day– old bees collected in August (14Summ02), and the brood nest bees collected in the winter and spring (BNBSpring) (*p* < 0.0014). However, there were no significant differences in phenylalanine levels between the newly emerged bees (NEBSumm01 and NEBSumm02), 28-day– old bees collected in August (28Summ02), and the brood nest bees collected in the fall (BNBFall; *p* > 0.05). Markedly higher levels of tyrosine were observed in the newly emerged bees collected in June and July (NEBSemm01 and NEBSumm02) compared to the other bees (H_(8)_ = 99.77, *p* < 0.0001). Higher levels in brood nest bees collected in the fall (BNBFall) were also observed and were significantly different from 28-day– old summer bees collected in July as well as the winter brood nest bees (28Summ01 and BNBWinter, respectively; *p* < 0.0014). Similar results were observed for tryptophan (H_(8)_ = 76.03, *p* < 0.0001). β-alanine showed a different pattern with the 14-day– old bees (14Summ01 and 14Summ02), having higher levels compared to most of the other metabolites (H_(8)_ = 120.40, *p* < 0.0001), except for the 28-day– old bees collected in August and the brood nest bees collected in spring (28Summ02 and BNBSpring). A similar trend was observed for sn-glycerol-3-phosphocholine, where higher levels were observed in the 14-day– old bees (H_(8)_ = 61.45, *p* < 0.0001). The intermediate of phosphatidylcholine synthesis, O-phosphocholine, occurred at lower levels in 28 day– old bees(28Summ01 and 28Summ02) compared to the rest of the metabolites (H_(8)_ = 61.45, *p* < 0.0001), with significant differences observed for the 14-day– old bees collected in July (14Summ01; *p* < 0.0014). However, choline was at significantly lower levels in newly emerged bees (NEBSumm01 and NEBSumm02) compared to the rest of the metabolites (H_(8)_ = 107.85, *p* < 0.0014), with the exception of the 28-day– old bees collected in August and the brood nest bees collected in the fall (28Summ02 and BNBFall; *p* > 0.0014). Levels of choline in 28-day– old bees sampled in July (28Summ01) were higher compared to the newly emerged bees (*p* < 0.0014) and the brood nest bees collected in the fall (NEB01, NEB02, and BNBFall, respectively, *p* < 0.0014). In addition, there were higher levels of proline in the 14-day– old bees (14Summ01 and 14Summ02) compared to the rest of the metabolites (H_(8)_ = 105.33, *p* < 0.0001), and this was similar to AMP, although higher levels were observed in 14-day– old summer bees collected in July (14Summ01) (H_(8)_ = 99.13, *p* < 0.0001). Differences in metabolite levels were indicative of differences in the metabolism of honey bee brains depending on age, but there were also indications that time of collection over the year was a factor. Apart from the significant differences in metabolite concentrations for the nine VIP scores >1, all of the other 21 metabolites showed significant differences between some timepoints (Supplementary Table S5), which indicates that they might also reflect to differences in the metabolic composition of healthy bee brains as they age.

## 4. Discussion

The largest group of compounds out of the 30 metabolites identified in the bee brain were related to amino acids. This is similar to results from McDevitt et al. [34], who reported the detection of 30 to 35 metabolites in bee brains, as well as Pizzorno et al. [13], who detected 25 metabolites by NMR in bee brains with ten amino acid/amino acid intermediates or amino acid derivatives. Many of the metabolites of those studies were the same as the ones found in this study, including acetate, alanine, aspartate, choline, glutamate, glutamine, glycine, O-phosphocholine, proline, sarcosine, succinate, taurine, trehalose, sn-glycero-3-phosphocholine, and β-alanine. However, McDevitt et al. [34] focused on differences between the castes (drones and worker bees) and tissues (brain, head, and whole body), while Pizzorno et al. [13] focused on the effect of the deformed wing virus infection on the metabolome of bee brains. In this study, nine VIPs (>1) were significantly different when comparing bee brains of different ages and times of the year. These were four protein amino acids (β-alanine, phenylalanine, tryptophan, and tyrosine), one proteinogenic amino acid (proline), three choline/choline derivatives (choline, O-phosphocholine and sn-glycero-3- phosphocholine), and one nucleotide (AMP). In contrast, the brains of worker bees either injected with deformed wing virus, not injected, or mock injected only had significantly different proline levels, with an increase in following virus injection [13]. This indicates that aging may have different impacts on the brain than a virus infection.

A comparison of the nine VIPs in summer bees revealed that the results were relatively similar between bees sampled in mid-summer (June to July) with those sampled in late summer (July to August). The greatest changes were observed in the transition from newly emerged bees to 14-day– old summer bees than from 14-day– old to 28-day– old summer bees with β-alanine, sn-glycero-3-phosphocholine, proline, AMP, and O-phosphocholine showing a consistent decline as adult bees aged from 14 to 28 days.

For overwintering brood nest bees, several patterns were also observed for the nine VIPs. Phenylalanine, tryptophan, and tyrosine had almost identical patterns with a drop between fall and winter, remaining low in spring, but choline, sn-glycero-3- phosphocholine, proline and AMP were relatively unchanged between fall, winter, and spring. β-alanine showed an increase from fall to winter and again in spring, while O-phosphocholine remained relatively unchanged between fall and winter, followed by a drop between winter and spring. Thus, the only metabolite reflective of a progressive stress of placing bees in storage over time (i.e., a continuous change from fall to winter to spring) was β-alanine, which kept increasing.

Phenylalanine, tyrosine, and tryptophan had very similar patterns in both summer and overwintering bee brains, decreasing in the newly emerged bees to the 14-day– old summer bees and in the fall to winter brood nest bees. This could be important to bee function as phenylalanine and tyrosine are precursors of the neurotransmitters, dopamine and octopamine [35,36], and tryptophan is a precursor of the neurotransmitter, serotonin [37]. Dopamine is involved in cognitive processes, such as learning and memory, as well as behaviors like aggression [38], while octopamine mediates responses of stresses and behaviors like flight, aggression, escape, response to sucrose, and division of labor [39]. Serotonin has been linked to neural functions and the response to stimuli, like defensive behavior [40]. Similar to the 28- and 14-day– old bee comparison in this study, serotonin levels were reported to increase in the mushroom bodies of the forager bees compared to the nurse bees, but that did not correspond with differences in behavior associated with precocious foraging or the reversion of foraging behavior [41]. The changes in these three amino acids may be related to the 14-day– old bees being involved in tasks that require the performance of more complex neural and cognitive processes than newly emerged bees, like hygienic behavior, cell cleaning, capping brood, and tending to the brood and the queen [17]. In the fall, brood nest bees typically perform similar tasks related to nest maintenance but survive much longer [42]. Both 14-day– old bees and fall brood nest bees are less likely to switch to other tasks, which may explain their similar metabolic profiles. Therefore, a decrease in phenylalanine, tyrosine, and tryptophan could be related to a higher demand for these amino acids to synthesize neurotransmitters. Changes in phenylalanine, tyrosine, and tryptophan, however, appear less likely to indicate late aging and physiological changes due to seasonality, as no significant further declines were observed from the 14- to 28-day old summer bees or winter to spring brood nest bees. This is not surprising as cognitive demands, based on the tasks performed, could be similar between the 14- and 28-day old bees, and between winter and spring bees. However, one might expect more demand for neurotransmitters in the 28-day– old bees as they can be involved in complex tasks, like patrolling and foraging [17]. Tasks like foraging demand learning, information processing, and memorizing [43]. Similar to summer bees, brood nest bees in the fall might have more demands for neurotransmitters, as they will be tending to the brood, performing hygienic tasks, processing nectar and pollen, and defending the nest from invaders [17]. From winter to early spring, however, they would continue some of the same tasks, but mostly tending to the queen and regulating temperature. One possibility is that sufficient neural connections have been established in 14-day– old bees and fall brood nest bees that the levels of phenylalanine, tyrosine, and tryptophan can remain stable.

The patterns of three intermediates in the synthesis of the neurotransmitter acetylcholine, namely sn-glycero-3-phosphocholine, O-phosphocholine, and choline [44], revealed an increase in the newly emerged bees to the 14-day– old in summer bees (except for one set of summer bees for O-phosphocholine). Acetylcholine is the main excitatory neurotransmitter of the central nervous system of insects and is involved in a wide range of associative and non-associative learning, such as that required for orientation flights, foraging trips, and scouting trips [45]. Increases in sn-glycero-3-phosphocholine, O-phosphocholine, and choline could be related to changes in neural functions needed after the bees emerge, as described above for several amino acids, except, in this case, these changes may be related to decreased levels of the neurotransmitter acetylcholine as those compounds are associated with the main behaviors of the 14-day– old bees. While choline did not further decline, sn-glycero-3-phosphocholine and O-phosphocholine levels were reduced from 14 to 28 days, which could be linked to a change in the behaviors of older bees, such as orientation and foraging, that could require more acetylcholine, resulting in a higher consumption of intermediates. The difference in O-phosphocholine between the newly emerged bees and the 14-day– old bees in mid- and late summer, with an increase versus no change, respectively, suggests that O-phosphocholine is more sensitive to age and the environment. For brood nest bees, declines in fall to winter for only sn-glycero-3-phosphocholine, and choline could be related to the cognitive demands at that time, which may be different from winter to spring, when only the O-phosphocholine levels decrease. However, the interactions and demands of these compounds are more complex than explained here. For example, choline has several roles in cells in addition to being a part of the synthesis of acetylcholine, including acting as major component of membrane phospholipids, and as source of methyl groups to form S-adenosylmethionine [46].

Proline is involved in many processes, including glucogenesis and lipogenesis [47]. Its involvement in lipid formation could partially explain its similar pattern of change to that of choline. Proline is present in the insect hemolymph and brain [48,49]. Pizzorno et al. [13] proposed that increased proline in the brains of bees infected with deformed wing virus was related to an increase in the antimicrobial peptides (AMPs), abaecin, and apidaecin. Perhaps the increase in abaecin and apidaecin in 14-day– old bees could be related to a need for increased immunity of the brains at that age, while bees are performing hygienic and grooming behaviors [50,51]. As proline is one of the most abundant proteinogenic amino acids found in nectar [52], its increase in 14-day– old bees could be at least partially explained by the greater consumption of nectar after bees have emerged. This would not change as much between 14- and 28 day– old bees, which may also explain why its levels remained relatively unchanged in older bees. Additionally, the oxidation of proline can enhance carbohydrate metabolism, which may enhance thermotolerance in insects [53]. Perhaps the decline in proline during the fall is due to an increased demand as part of the initial adaptation of bees to colder temperatures through proline oxidation. However, this hypothesis warrants further investigation.

In summer, β-alanine levels showed a similar pattern as proline, increasing in 14-day– old bees and then remaining unchanged. β-alanine appears to be a neurotransmitter that is structurally intermediate between α-amino acid (glycine, glutamate) and γ-amino acid (GABA) neurotransmitters [54]. β-alanine is found in the central nervous system and hemolymph of insects [48,55] and is involved in several unique biochemical processes in insects, including the inactivation of photoreceptive histamine and the regulation of circadian rhythms by conjugation with dopamine [56,57]. The consumption of proline and β-alanine seems to have similar effects on insect behaviors, like inhibition of nest construction by *Vespa orientalis*, as well as a reduction in its lifespan [49]. Ingestion of β-alanine also affected the longevity and behavior of *Bombus terrestris*, such as increased walking instances and lowered syrup consumption, but it did not influence the longevity or walking behavior, even though treated honey bees showed lower feeding indices compared to the control [58]. β-alanine is an abundant non-protein amino acid found in nectar and honey [52,59], and thus its increase in 14-day– old bees could be due to the consumption of nectar by that age, similar to proline. β-alanine was unique among the VIPs in that it progressively increased from fall to winter and then winter to spring in the brood nest bees. In mammals, its accumulation can delay brain development and cause oxidative stress thus disturbing energy metabolism [60]. Unlike mammals, however, honey bees do not show signs of senescence as they get older, such as winter bees not showing a decline in learning and discrimination abilities, although some decline in associative learning has been reported [24]. Therefore, while β-alanine accumulation in overwintering bees over time could be negative, it appears unlikely as β-alanine levels in brood nest bees measured in spring were still well below those measured in relatively young 14-day– old summer bees.

The pattern of AMP levels in summer bees was like those of proline and β-alanine with an increase in 14-day– old bees, followed by little change in the 28-day– old bees. Adenosine monophosphate has many functions in organisms. It can regulate cellular functions, such as cell growth and differentiation, gene transcription and protein expression [61]. Increases in AMP/ATP ratio can activate adenosine monophosphate-activated protein kinase, which is an important energy sensor, maintaining cellular energy homeostasis, whose dysfunction can result in cellular stress associated with aging [62]. An imbalance in oxidative phosphorylation, including an increase in the AMP/ATP ratio, has been observed in aging mice [63]. This pattern could be related to high demands on energy requirements linked to the complex tasks that the bees perform. However, this may not be the case in this study, as an increase in AMP was not observed in the 28-day– old bees, nor at any time point for brood nest bees.

In summary, all the metabolites with significant differences between the samples of this study could have direct or indirect effects on bee brain function. However, many of the metabolites that significantly changed in the bees of different ages, in summer and at different timepoints over winter, were be related to the synthesis of neurotransmitters (i.e., dopamine, octopamine, serotonin, and acetylcholine), and are possibly necessary for the performance of complex behaviors. Even though most of the changes in the bee brain metabolome were related to amino acids, several of them also have functions as neurotransmitters. The decrease in β-alanine, proline, sn-glycero-3-phosphocholine, O-phosphocholine, and AMP between the 14- and 28-day– old summer bees could be related to aging, whereas no metabolites significantly increased over that time period. In contrast, in overwintering bees, β-alanine increased progressively, proline and sn-glycero-3-phosphocholine declined in fall, O-phosphocholine declined in spring, and AMP remained unchanged from fall to spring. Therefore, those same metabolites had a diverse set of concentration patterns over winter. The only marker for aging in both summer and winter that declined late in the life cycle of the bees was O-phosphocholine, although β-alanine also changed later in the life cycle, but with an inverse response in summer versus winter. Thus, potential markers for the effect of stressors on bee health could be linked to O-phosphocholine and β-alanine concentrations. While Lee et al. [20] compared the metabolites of whole bodies of winter and summer bees, they only examined one time point for each season. This study shows that metabolite levels differ from bees collected in the summer and winter, emphasizing the need to sample multiple times as well sampling specific tissues. Although the mid-summer (June to July) and late summer (July to August) samples showed very similar patterns, there were some differences, indicating that even summer bees may vary depending upon the time of that season. In addition, this study shows that the metabolites in the brain of bees notably change over time and thus perhaps studies such as Pizzorno et al. [13], that examined the metabolome of the brains of bees infected with deformed wing virus, or Lee et al. [20], that used a single time point to compare the metabolites in whole bee bodies in summer and winter, may want to include bees of different ages when comparing the effect of biotic stressors or storage. This study provides a baseline of the metabolome of healthy bee brains at different ages and seasons, which should help future studies to design sampling strategies to assess the effect of stressors on bee health and longevity.

## Figures and Tables

**Table 1:** Schedule for marking and collecting worker honey bees for NMR analysis. NA = not applicable.

NEB Marking and Collection	14-Day– Old Bee Collection	28-Day– Old Bee Collection	Brood Nest Bees	Number of Colonies
24 June 2019 (NEBSumm01)	8 July 2019 (14Summ01)	22 July 2019 (28Summ01)	NA	30
24 June 2019 (NEBSumm02)	7 August 2019 (14Summ02)	21 August 2019 (28Summ02)	NA	28
NA	NA	NA	17 September 2019 (BNBFall)	26
NA	NA	NA	24 February 2020 (BNBWinter)	17
NA	NA	NA	8 May 2020 (BNBSpring)	15

## Data Availability

Data are contained within the article or Supplementary Material.

## Supplementary Materials

The following supporting information can be downloaded at: https://www.mdpi.com/article/10.3390/insects15080578/s1. Supplementary Table S1. Metabolite concentrations (mM). Metabolite concentrations (mM) of newly emerged bees collected in the summer (NEBSumm01 and NEBSummer02), 14-day– old worker bees collected in the summer (14Summer01 and 14Summer02), 28-day– old worker bees collected in the Summer (28Summer01 and 28Summer02), brood nest bees collected in the fall (BNBFall), winter (BCBWinter), and spring (BNBSpring). Supplementary Table S2. Variable importance in projection (VIP) scores. Data of the Variable of importance in projection (VIP) scores for the 30 identified metabolites, and the chemical indicator (DSS-d6), based on a Partial Least Squares–Discriminant Analysis (PLS-DA). Supplementary Table S3. Descriptive statistics of metabolite concentration of the nine VIP (>1). Descriptive statistics of proline, tyrosine, β-alanine, sn-glycerol-3-phosphocholine, phenylalanine, tryptophan, AMP, choline, and O-phosphocholine concentrations (mM) in brains of newly emerged bees collected in the summer (NEBSumm01 and NEBSummer02), 14-day– old worker bees collected in the summer (14Summer01 and 14Summer02), 28-day– old worker bees collected in the Summer (28Summer01 and 28Summer02), brood nest bees collected in the fall (BNBFall), winter (BCBWinter), and spring (BNBSpring). Supplementary Table S4. Corrected *p*-values of multiple pairwise comparisons using Conover-Iman procedures after a significant Kruskal–Wallis tests for the nine VIP (>1), with a Bonferroni corrected significance level of 0.0014. Supplementary Table S5. Results of Kruskal–Wallis tests using ranked transformed data, followed by multiple pairwise comparisons using Conover–Iman procedures and Bonferroni corrections procedure to confirm if age and season affected the concentration of the 30 metabolites identified in honey bee brains.
